# Contextualising the microbiota–gut–brain axis in history and culture

**DOI:** 10.1080/16512235.2019.1546267

**Published:** 2018-11-13

**Authors:** Alison M. Moore, Manon Mathias, Jørgen Valeur

**Affiliations:** School of Humanities and Communication Arts, Western Sydney University, Penrith, Australia; School of Modern Languages and Cultures, University of Glasgow, Glasgow, UK; Unger-Vetlesen Institute, Lovisenberg Diaconal Hospital, Oslo, Norway

This special edition on humanistic approaches to the microbiota–gut–brain axis was inspired by two symposia organised by literary scholar Dr Manon Mathias at the University of Aberdeen in 2017 and at the University of Glasgow in 2018, both involving the participation of medical historian Dr Alison M. Moore and gastroenterological researcher Dr Jørgen Valeur, with all the contributors to this special edition having spoken at one or other symposium. Mathias and Moore are among the rare cohort of humanities scholars who approach past literary, cultural and medical concepts with the aim of contextualising current medical models and research findings, while Valeur is among the even rarer cohort of medical researchers and clinicians to see inherent value in humanistic understandings of health. It is the shared premise of all three editors that historical and cultural perspectives enrich the current understanding of microbial ecology, and the science of microbe–host interactions.

One reason it should interest all medical researchers and clinicians to read the articles in a special edition such as this, is to consider what is truly novel in current scientific models and what may be inherited from past medical concepts. Such earlier concepts may help or hinder current science, but without researchers knowing anything about them, it is most likely that their influence will not be helpful. As the American enteric nervous system researcher Michael D. Gershon noted in his 1998 book on the *Second Brain*, ‘hubris for scientists comes from inadequate knowledge and appreciation of the past’ [1]. Indeed, failures to see what is truly new in the treatment of gastroenterological disorders can be found throughout the scientific record.

The history of faecal microbial transplant (FMT) is a case in point: though often claimed as a ‘new’ therapy [2], it has existed in the form of oral administration in European medical traditions since Ancient Greece, featured in several major works of medical description of the sixteenth and seventeenth centuries [3], and has been used in Chinese medical traditions since the Don-jin dynasty (4^th^ century CE) [4]. Rectal delivery of FMT was used by the American doctor I.O. Wilson in 1910, following the identification of changes in faecal bacterial composition among patients with functional bowel disorders [5]. Thus, FMT is anything but ‘new’ and its historical and trans-cultural ubiquity may indeed lend support to the emergent scientific model of the gut microbiome as an essential organ of the human body, composed of organisms that have co-evolved with our own cells such that they are to some extent ‘us’. This indeed is the very argument that FMT researcher Alexander Khoruts has made for why this therapy for Clostridium difficile should be seen not as a ‘drug’ but as a ‘transplant’ [6]. The mounting evidence for commensal and symbiotic intestinal microbes lends itself to this interpretation, and is consistent with the acceptance of the microbial origin of our cellular mitochondria [7].

It is not hard to see then why new research on the gut microbiome should fascinate scholars in the humanities since it touches upon the very question of what it means to be human – indeed the core concern of these disciplines. Humanities scholars are richly imaginatively endowed, as both Bencard & Whiteley’s and Lucas’ creative endeavours in exhibiting and narrativising medical research on the microbiota–gut–brain axis demonstrate. They are also particularly trained in critical and contextual ways of reading concepts, a skill-set generally missing from science degrees, as Moore herself was surprised to discover when studying biomedical sciences at an Australian university between 2010 and 2013. A 2018 article involving two biomedical researchers, Katarzyna Hooks and Jan Peter Konsman, with the philosopher of microbiology Maureen O’Malley, offering a critical evaluation of microbiota–gut–brain research, is an excellent case in point [8]. The authors, while acknowledging the importance of microbiota–gut–brain axis research for understanding brain function and behaviour, show that there are frequent weaknesses in study design and conceptual modelling in the field, as well as in public communication, with pre-emptive hyperbole too often capturing popular health movements. But we might do well to remember also that medical research does not only filter out into popular cultural imaginaries, but is indeed situated within specific cultural and historical contexts. Our special edition is precisely about some of the earlier medical concepts that have helped to prime medical researchers toward posing questions about the brain by turning to the gut, and about how current medical research on the microbiota–gut–brain axis can be responsibly publicly disseminated.

The paper that engages in most depth with the question of scientific dissemination and public engagement is Bencard & Whiteley’s piece on the ‘Mind the Gut’ exhibition at the Medical Museion, Copenhagen. This exhibition was the result of intense reflection involving a range of academic researchers, artists and curators, and the article reveals the importance of this extended dialogue that took place over two years before the exhibition itself was launched. For example, one of the outcomes of the discussions was an increased awareness of science as process, hence the decision to display projects as they progressed rather than a complete set of data. The inclusion of this article is important as makes it clear to the wider scientific community what can be achieved when different groups come together to engage in deep reflection on how to engage public audiences. The need for such endeavours is now greater than ever before in our digital age and with the emergence of popular science communicators and journalists. The public now has tremendous capacity to access – but not necessarily to understand the nuances and limits of – scientific research on health and what it may mean for individuals. Information is not all that is needed either – the humanities and creative arts can most certainly help to inspire.

While most of the papers in this volume touch upon the question of how a connection came to be made between the mind and the gut in history and culture as a precursor to the current concept of the microbiota–gut–brain axis, not all these papers show an explicit connection to questions of microbial ecology. The microbiota–gut–brain axis is indeed a quite recent innovation and is not to be found in the nineteenth-century configurations described here. As Peter Down noted in his *History of Luminal Gastroenterology in Britain*, the brain historically was most often thought to connect to the stomach rather than the colon that is most implicated in current microbiota–gut–brain research today, since the colon was generally viewed ‘as a tube that merely stored and evacuated the waste products of digestion’ [9]. In the words of one early twentieth-century British surgeon, the colon was ‘simply a sewer canal’ [10].

However, the discovery of microorganisms at the end of the nineteenth century did impact one important area of mind–gut consideration, fuelling the pre-existing concept of ‘autointoxication’ – a topic discussed in the papers by Mathias, Lillestøl and Moore in this special issue. Early configurations of this concept viewed constipation as dangerous because it was thought that toxic biproducts of digestion were absorbed into the blood, causing a systemic poisoning of the body which included the brain and the nervous system. Microbes had been found *in vitro* to putrefy animal and vegetable material, so it was assumed that they also did so in the colon, providing mechanistic support to the theory of autointoxication [6]. As Mathias’ paper in this volume shows, both German and French physicians in the late-nineteenth century indicated that microbes might be responsible for the autointoxication they ascribed to constipation, and in 1887, the French physician Charles Bouchard proposed microbial imbalance as the cause of several diseases he saw as resulting from autointoxication. Mathias also suggests why these ideas were so widespread in France, especially in relation to mental distress, and why they became discredited in twentieth-century scientific research – in part because popular uptakes of autointoxication by purveyors of herbal remedies and enemas to relieve constipation, as well as by evangelical diet gurus such as the American John Harvey Kellogg, reduced the reputation of the theory by associating it with widespread quackery.

Autointoxication formed part of several late-nineteenth-century disease categories, from dyspepsia, discussed in Miller’s paper, to *neurasthenia gastrica*, examined in Lillestøl’s paper. Moore’s paper shows how a mind–gut connection came to support the late-nineteenth-century psychiatric description of coprophagia as both a sign of mental illness, and as a suspected cause of it. But microbes remained under-appreciated in this early body of scientific psychiatric thought, and indeed even the most recent medical investigations of institutional coprophagia have not fully explored the potential microbial interactions entailed in it.

Nineteenth-century ideas about the mind–gut connection tended to assume that the relationship was bidirectional, something discussed in the papers of Mathias, Miller, Moore, Lillestøl and Lucas. Lillestøl reveals an emerging interest for interactions between the central nervous system and the gastrointestinal tract throughout the nineteenth century; early descriptions of a field that we today would label as neurogastroenterology, and diagnoses that we now denote as functional gastrointestinal disorders. And as Miller shows, dyspepsia was considered both a disorder of the stomach and of the mind, underpinned by the concept of ‘nervous sympathy’, which pre-empted the later discovery of the enteric nervous system as a mechanism through which the viscera communicated nervous signals to the brain. Miller notes that this older holism was overturned through twentieth-century forms of anatomical, physiological and surgical scholarship which tended to isolate the stomach as more was discovered about it; but the holism is found again in the new model of the microbiota–gut–brain axis. Gershon too referred to the twentieth-century medical insistence on a one-way direction in which patients with unexplained gastroenterological symptoms were viewed as ‘hypochondriacs’, situating his own research on the enteric nervous system in both a more holistic and a more patient-centred approach [1]. Indeed, in 1977, the British psychiatrist Peter Dally insisted that all patients presenting to a gastroenterology clinic whose symptoms could not be ascribed to ‘an organic cause’ must be suffering from a psychiatric, not gastroenterological illness [9]. In this model, functional disorders were thought to be caused primarily by the patient’s psychic distress. As Lucas’s paper in this volume shows, the emerging evidence of the role of microbial ecology in mental health is a force against this one-way paradigm by providing a mechanism to explain how the gut in turn influences the brain. But as Miller argues, this holism, in and of itself, is anything but new.

Are there lessons to be gained from the past for current microbiota–gut–brain axis researchers? Perhaps these might be summarised as follows: (1) Speak not of what is ‘new’ before knowing what is old; (2) recognise the power of popular cultural uptakes of science in shaping what new generations of scientists both absorb and react against; and (3) work with the humanities and creative arts to build a more science-conscious public awareness that accurately reflects the findings of microbiota–gut–brain research.
